# Practical issues regarding implementing a randomized clinical trial in a homeless population: strategies and lessons learned

**DOI:** 10.1186/s13063-017-2046-9

**Published:** 2017-07-05

**Authors:** Olamide Ojo-Fati, Anne M. Joseph, Jed Ig-Izevbekhai, Janet L. Thomas, Susan A. Everson-Rose, Rebekah Pratt, Nancy Raymond, Ned L. Cooney, Xianghua Luo, Kolawole S. Okuyemi

**Affiliations:** 1 Department of Family Medicine and Community Health, University of Minnesota Medical School, 717 Delaware St. SE, Suite 166, Minneapolis, MN 55414 USA; 2 Program in Health Disparities Research, University of Minnesota Medical School, 717 Delaware St. SE, Suite 166, Minneapolis, MN 55414 USA; 3 Department of Medicine, University of Minnesota Medical School, 717 Delaware St. SE, Suite 166, Minneapolis, MN 55414 USA; 4 Department of Psychiatry, University of Minnesota Medical School, 2450 Riverside Ave., F282/2AW, Minneapolis, MN 55454 USA; 5 Department of Psychiatry, Yale University School of Medicine, 300 George St., Suite 901, New Haven, CT 06511 USA; 6 Division of Biostatistics, School of Public Health, University of Minnesota, 420 Delaware Street SE, Minneapolis, MN 55455 USA; 7 Biostatistics Core, Masonic Cancer Center, University of Minnesota, Minneapolis, MN 55455 USA; 8 Department of Family & Preventive Medicine, University of Utah, 375 Chipeta, Suite A, Salt Lake, UT 84108 USA

**Keywords:** Community-based, Clinical trials, Homeless adults, Attrition, Smoking cessation, Retention

## Abstract

**Abstract:**

There is a critical need for objective data to guide effective health promotion and care for homeless populations. However, many investigators exclude homeless populations from clinical trials due to practical concerns about conducting research with this population. This report is based on our experience and lessons learned while conducting two large NIH-funded randomized controlled trials targeting smoking cessation among persons who are homeless. The current report also addresses challenges when conducting clinical trials among homeless populations and offers potential solutions. Homeless individuals face several challenges including the need to negotiate daily access to food, clothing, and shelter. Some of the critical issues investigators encounter include recruitment and retention obstacles; cognitive impairment, mental health and substance abuse disorders; transportation and scheduling challenges; issues pertaining to adequate study compensation; the need for safety protocols for study staff; and issues related to protecting the wellbeing of these potentially vulnerable adults. Anticipating realistic conditions in which to conduct studies with participants who are homeless will help investigators to design efficient protocols and may improve the feasibility of conducting clinical trials involving homeless populations and the quality of the data collected by the researchers.

**Trial registration:**

ClinicalTrials.gov, ID: NCT00786149. Registered on 5 November 2008;

ClinicalTrials.gov, ID: NCT01932996. Registered on 20 November 2014.

## Background

The annual number of persons experiencing homelessness in the United States is estimated to be approximately 3.5 million, and this number is increasing [1, 2]. The rate of homelessness is increasing due to both individual challenges (e.g., poverty, mental health and substance use disorders) [3, 4], and structural factors (e.g., lack of affordable market-rate housing, low-wage jobs, diminishing housing voucher program (Section 8), and changing subsidized housing eligibility guidelines) [5]. Homeless individuals experience higher rates of physical and mental illness leading to higher rates of hospitalization and mortality compared to those in the general population [6].

Homeless individuals have a mortality rate three times higher than the general population [7]. One study showed that the average homeless person has a life expectancy of only 47 years, compared to an average of the general population, 77 years [8]. Many factors may contribute to the high mortality rates among homeless adults, including affective disorders [9], chronic illnesses (e.g., HIV, heart disease, and cancer) [10], and excess alcohol and drug use [11]. This population also has poor medication adherence and low self-efficacy which, along with the many practical challenges of homelessness, limits the ability to adopt behaviors that lead to better health outcomes [12]. Several studies show that homeless individuals in the United States have elevated rates of chronic illnesses such as human immunodeficiency virus (HIV) infection, tuberculosis (TB), and mental health and substance use disorders [13–15]. Approximately 33% of adults who are homeless suffer from some form of severe and persistent mental illness [16] as compared to the general population estimates of 18.1% [17]. These findings highlight the need for clinical research to identify effective interventions to improve health and prevent disease in this vulnerable and underserved community [18]. Despite the high prevalence of medical and mental health illnesses in this population, very few intervention studies are conducted among homeless adults [19] and little is known about effective interventions to address both acute and chronic conditions in these vulnerable adults. Homeless individuals are generally excluded from research studies because they are perceived as “hard to reach and retain” [20–22]. This assumption stems from the fact that this population is often transient which may lead to high study attrition [23]. While this is true in some cases, some studies have demonstrated that high retention can be achieved in programs tailored to address the unique challenges inherent in conducting research among persons who are homeless [23, 24].

However, high attrition rates due to participant withdrawal, relocation, and death can threaten the integrity of study results which may contribute to researchers’ reluctance to engage with homeless populations. In order to decrease attrition, increase protocol adherence, and to protect study staff and homeless participants, it is important to address the issues that are germane to homeless populations.

### Study context

The first smoking-cessation randomized controlled trial (RCT) designed for homeless smokers was titled “Power To Quit” (PTQI) [24]. The PTQI study was a community-based RCT that enrolled 430 adult smokers who were homeless. PTQI compared Standard Care (one-time brief advice to quit smoking) to six Motivational Interviewing (MI) counseling sessions. The details of the study design and recruitment procedures have been published [25]. All participants received 21-mg nicotine patches for 8 weeks. Assessments were conducted at post-randomization weeks 8 and 26. Overall, this study found that cotinine-verified 7-day quit rates were 9.3% for MI and 5.6% for control at 26 weeks (*p* = 0.15) [24]. These quit rates are low compared to the general population and may be associated with the observation that many participants reported concurrent heavy alcohol use (approximately 46%). The results of a systemic review and meta-regression analysis show that the prevalence of alcohol dependence in the homeless population is 8.5–58.1% (*β* = 0.18, SE[*β*] = 0.07, *p* = 0.007) [5]. Therefore, a more intense follow-up intervention was designed to address some of the limitations encountered in the first trial and to target concurrent alcohol abuse.

PTQII included (1) a higher dose of pharmacotherapy (nicotine patch combined with nicotine gum or lozenge) and a longer duration of prescribed use (i.e., 12 versus 8 weeks), (2) an increased number of counseling sessions (12 versus 8 sessions over 3 months), (3) an increase in the duration of individual sessions from 15 to 30 min to 45 to 60 min, (4) counseling sessions utilizing cognitive behavioral therapy (CBT) strategies to enable counselors to provide more strategies during counseling sessions, and (5) counseling content targeting alcohol abstinence in addition to smoking cessation. Details regarding the design of this study have been published [26]. Briefly, the study utilizes a three-group design that includes (1) Usual care (UC) for smoking and alcohol cessation, (2) Intensive smoking cessation plus UC alcohol abstinence counseling (IS), and (3) Integrated Intensive Smoking and Intensive Alcohol Counseling (IntS + A). All participants were invited to receive 12 weeks of nicotine replacement therapy utilizing both nicotine patches (tailored to their baseline cigarettes smoked per day), plus their choice of nicotine gum or lozenge. Integrating alcohol treatment with the intensive smoking intervention helped to assess whether addressing alcohol use concurrently with smoking cessation will result in improved smoking abstinence and/or reduced alcohol use, an important scientific question that has never been studied in homeless populations.

Both PTQI and PTQII were approved and monitored by the University of Minnesota Institutional Review Board. The objective of this paper is to describe several practical lessons learned while conducting two large RCTs targeting smoking cessation among persons who are homeless. Our intention is to provide information that may assist other investigators who are interested in developing and testing the efficacy of interventions designed for homeless populations. Both studies were funded by the National Institutes of Health (USA).

The critical issues addressed in this paper are organized into four domains: (1) study settings, (2) participants, (3) data collection and management, and (4) staffing issues. For each domain we identify the central challenge, discuss the strategy used to address it, and highlight the potential implications for future research trials.

#### Study settings

##### Multisite studies

During the planning phase of both studies, a Community Advisory Board (CAB), consisting of program directors and managers from shelters and social service agencies, was formally established and convened twice a year. The CAB’s main function was to review the treatment materials, recruitment strategies, and counseling methods. They acted as advisors for the project. In addition, the CAB provided information on potential shelters and served as a resource for generating some of the strategies that the study used to manage recruitment of participants from multiple sites. In both trials, larger shelters in a large metropolitan city were first targeted to reach the greatest number of individuals. Sites with zero-alcohol policies were generally avoided in the PTQII study because this policy may discourage participants from being truthful about their alcohol use. It is necessary to include multiple sites to recruit an adequate sample for a clinical trial. However, different homelessness shelters may be structured in ways that introduce variability in the study implementation. For example, there may be different policies regarding alcohol and tobacco use, the availability of support services such as medical and mental health clinics, and other social support services.

##### Capacity to conduct work onsite

In PTQI and PTQII the study coordinator established contact with the prospective sites with short presentations to the shelter administrators detailing the purpose of the study, benefits to the community and the shelter. Given the personnel shortages and limited resources of many shelters, the studies aimed to make minimal demands outside of private space to administer surveys and to conduct counseling. On occasion, study participants may be banned from shelters for misconduct. This posed a problem for follow-up and required extending communication efforts and meeting places outside the shelter. The most successful way to access such participants was to contact the participant via phone or through other contacts provided by the participant and secure a location proximal to the shelter that was both safe and could facilitate data collection and counseling. Establishing and maintaining a good relationship with shelters’ staff is essential in conducting research at a community site.

##### Availability of onsite mental health and other support resources

**Fig. 1:**
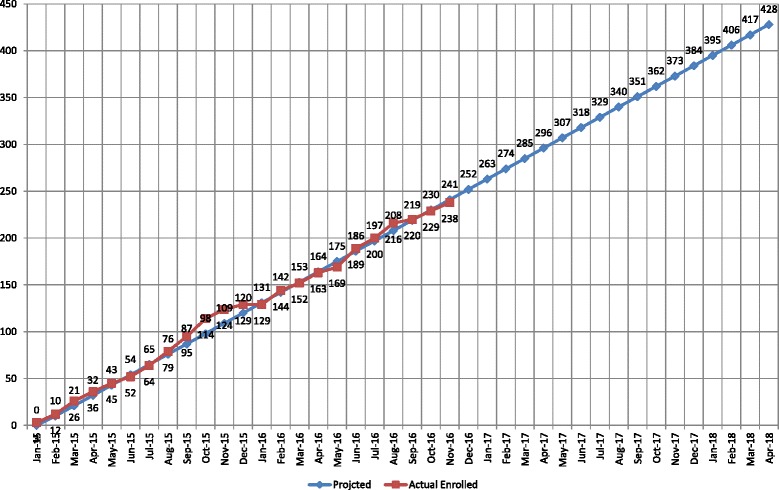
Target versus actual recruitment. Note: Among the 238 subjects, 156 are randomized to Usual Care and Integrated Smoking + Alcohol

In PTQI and PTQII we observed that selecting sites that offer mental health and other support services onsite facilitates study procedures. Some shelter sites have capacity for immediate referral to on-site licensed mental health providers for risk assessment. If a site does not have licensed mental health providers, a protocol to guide immediate decision-making by study personnel for appropriate referrals is needed. Such a protocol may include giving local resource center information to participants, and in the case of those in imminent risk, calling emergency medical services (EMS) or police. Ensuring that warm handoffs, a practice of directly linking patients with specialists, are made, even with EMS and police when possible, is important as there can be a history of distrust or challenging previous experiences between sites and services. Standard protocol is especially needed to involve the necessary authorities in referring suicidal participants. Staff should receive training on how and when to use security or police for safety back up (see the “Safety” section below). The study protocol of homelessness RCTs needs to address how staff should respond to participants in crisis; for example, with suicidal or homicidal ideation (see Fig. 1 for example).

##### Safety of study staff and participants

In both studies, we ensured that each study site had a specific protocol for how to handle participants who exhibit suicidal or homicidal behavior. Whenever participants displayed threatening behaviors, study staff immediately involved local security or the police if site security was not available. Additional safety measures included a two-person staffing (i.e., a buddy system) and scheduling study activities during times when shelter resources were readily available.

It was important to obtain prior consent from the participant for mandated reporting. Participants must be made aware of the limits of confidentiality when they enroll in studies, providing consent that clearly explains mandatory reporting. They should give verbal confirmation of understanding the limits of confidentiality at every study encounter. It was imperative to establish site-specific safety protocols for staff prior to initiating recruitment at each shelter site.

#### Study participants

##### Defining homelessness

It is important to use a robust definition of homelessness to establish participant eligibility for a clinical trial. The most widely used definition of homelessness is the Stewart B McKinney Act passed by the US Congress in 1987 which defines a homeless person as (1) any individual who lacks a fixed, regular, and adequate nighttime residence, and (2) one whose primary nighttime residence is a supervised publicly or privately operated shelter designed to provide temporary living accommodations, transitional housing including, but not limited to, emergency/overnight shelters with daily lottery, temporary transitional shelters, semipermanent, permanent transitional shelters, or other supportive housing program or a public or private place not meant for human habitation (e.g., on the streets or in abandoned buildings, tents, or automobiles) [27]. There are a number of alternative definitions – for example, “near homelessness” – that describe populations such as individuals living in wet/dry housing, a term used to describe an emergency housing for those who use alcohol. For the two RCTs described above, individuals living in campsites, vehicles, abandoned buildings or houses, parking garages, or on the street were considered homeless and thus eligible for participation in both trials. Excluded from our trials were those living in subsidized or Section 8 housing, those paying their own rent, and those who have been staying with a friend or family for more than 3 months.

##### Mental health eligibility criteria

RCTs frequently exclude participants with severe mental health or substance use problems; however, such exclusions would decrease the external validity of studies conducted among the homeless. Participants in the PTQ studies included those with major psychiatric disorders, provided they were stable and had no change in symptoms for the past 3 months; the ability to complete the consent procedure to participate in research and having no evidence of severe cognitive impairment. The seven-item Mini International Neuropsychiatric Interview (M.I.N.I.) [28], a well-validated scale, was administered by study staff at baseline and subsequent visits during the study to assess psychotic symptoms. The M.I.N.I. scale includes seven items such as the following: “In the past 30 days, have you believed that people were spying on you, or that someone was plotting against you, or trying to hurt you?”; “In the past 30 days, have you believed that someone was reading your mind or could hear your thoughts, or that you could actually read someone else’s mind or hear what another person was thinking?”; and “Do you ever hear things other people couldn’t hear, such as voices?” A M.I.N.I. score ≤5 was required for participants to be considered eligible for the study. Staff confirmed whether subjects with a score of 3–5 were receiving any psychiatric care, taking psychiatric medication, and experiencing any change in symptoms in the past 3 months. The principal investigator, in consultation with the study psychiatrist, made final decisions about eligibility in cases where concerns about a potential participant’s mental wellbeing or psychiatric status were raised. Participants with a score >5 on the M.I.N.I. scale were offered mental health resources, but were not eligible.

The Patient Health Questionnaire-9 (PHQ-9), a depression screening instrument [29], and the M.I.N.I. generalized anxiety disorder assessment [28], were also administered. Study staff used the Short Blessed Test to evaluate cognitive impairment that would make it both difficult and unethical to administer study materials [30]. Participants with score of ≥10 on the PHQ-9 scale and/or a positive score on the one suicidal ideation question were immediately referred to mental health services. Those with a score of ≥6 on the M.I.N.I. anxiety scale were probed for prior mental health evaluation in the last 90 days by a health care provider. Participants were encouraged to inform their mental health provider of their enrollment in a smoking-cessation trial, so that the providers can monitor symptoms and adjust their medications if clinically indicated. Under certain instances, it was medically and ethically necessary to give the participants referrals to additional community resources.

Due to the high prevalence of psychiatric comorbidity among homeless adults, a suicidality protocol (Fig. 1) was developed by the psychiatrist (co-investigator) on the research team in collaboration with the principal investigator and other co-investigators to guide risk assessment and respond to expressed suicidal ideation and intention. At the beginning of each eligibility interview, the following statement regarding mandatory reporting was given to each participant: “*I will keep all of the information you share with me confidential with one exception. If you give me information that indicates you are going to do something to hurt yourself or others in the near future, I will need to notify the proper authorities to make sure you and others are safe*.” This statement was included to ensure that participants understood the potential limits of privacy for their protection. Research staff screened participants for potential suicide risk using the Columbia Suicidality Severity Rating Scale (CSSR-S) (Fig. 1), a tool developed to train and certify unlicensed staff in administration [31]. All staff completed training in order to administer this instrument. An on-call system was established in the event that a participant screened for positive suicidal ideation.

##### Substance use eligibility criteria

PTQII specifically sought out participants who used alcohol. It was, however, important to consider impairment related to drug or alcohol intoxication, and potentially dangerous substance withdrawal symptoms. The revised Clinical Institute Withdrawal Assessment of Alcohol Scale (CIWA) [32] and the Alcohol Use Disorder Identification Test (AUDIT) [33] were used to evaluate alcohol withdrawal symptoms. Several studies have shown the reliability and diagnostic value of the AUDIT in screening for alcohol use disorders [33, 34]. The audit criteria for inclusion in our study was a score of between 7 and 26 because we were specifically treating those with alcohol and nicotine use problem. The CIWA is a useful tool for the assessment of risk for alcohol withdrawal. It was administered to participants with audit scores ≥19 to evaluate the extent of withdrawal symptoms and determine eligibility [35] based on the recommendations made by the developers of the AUDIT. Research staff were also trained to assess alcohol and drug-related adverse events and made the final determination regarding study continuation. At eligibility, persons with defined high AUDIT scores of ≥26 [33, 36] were excluded. A CIWA score of ≥8 or high withdrawal symptoms were also excluded because we determined they were likely too ill to participate in an outpatient treatment study. We offered these individuals referrals to alcohol treatment centers in the community.

Homeless adults are at a higher risk for alcohol and other recreational drug use than the general population [6, 10]. Therefore, excluding drug and alcohol users would limit the generalizability of study results for homeless populations. Careful consideration of safe and effective ways to include these individuals as study participants is warranted.

##### Recruitment

In PTQI and PTQII we observed variable recruitment by the time of month. For this reason, recruitment, assessment, and intervention sessions were scheduled around times when the homelessness shelters were the busiest. This included mealtimes that draw in those who were homeless and were coming for a meal but were not staying in the shelter overnight. In colder weather the overnight shelters had a high occupancy rate leading to higher recruitment rates. However, recruitment suffered a very significant reduction during summer months. During warmer months, outreach was intensified to recruit homeless individuals and establish recruitment in shelters sites. Recruitment also varied during certain times of the month. For instance, homeless individuals often received monetary government assistance which came in the form of checks typically issued on the first day of the month. As a result, during the first few days of the month potential participants were able to pay for a place to stay and were thus, less likely to occupy shelters at that time. Therefore, it was important to make an extra effort to remind participants of their appointments during those weeks. Although this phenomenon decreased recruitment at the beginning of the month, an opposite trend was observed at the end of the month when funds were less available and shelter occupancy increased.

In summary, seasonal conditions are important to consider when creating a schedule for visits to a site. The availability of homeless populations for study participation follows both seasonal and monthly trends. For this reason, studies done in this population should consider variability in participation depending on the time of the month in their scheduling [37].

##### Administration of informed consent

In PTQI and PTQII, participants completed a consent procedure and signed to be enrolled in the trial. The consent form language should consider the possibility of low educational attainment of persons who are homeless and should be written at the 5th-grade reading level so that the participants can fully understand the content of the consent process. Asking open-ended questions before the documents are signed is a recommended procedure by institutional human subject protection committees to ensure that participants understand the requirements of study participation. In PTQI and PTQII, these questions included *(1) Tell me what you understand about what will be required of you if you choose to be in this study? (2) What are the risks involved in quitting smoking? What are the risks involved in quitting drinking? and (3) What are the benefits of participating in this study?* In this vulnerable population extra care is needed and the entire process may take longer than usual. Provisions were made for participants who could not read by having the study staff read the entire consent form aloud. The decision about whether those with a legal guardian should be eligible for study participation should ultimately depend on the research topic and level of risk for participants in the study; however, inclusion may be appropriate [38]. In the PTQI and PTQII studies all subjects were legally competent to give consent.

##### Randomization

Although each participant was randomized individually in both studies, there was still potential for contamination of intervention assignment due to the community setting, where daily activities, such as eating and sleeping, may happen in a communal space. It is important to consider the relative advantages of individual versus group randomization for studies conducted in the setting of homelessness shelters because there is considerable communication between participants. Cluster randomization may be a robust approach to address this methodological issue but typically requires a larger sample size in terms of both the number of shelters and the number of individuals [39]. The application of cluster randomization could be appropriate in a larger-scale study in which there are more homelessness shelters participating if people within the same shelter are interacting with each other throughout the day.

### Intervention

In PTQI and PTQII we had limited availability of times to see participants at the shelters. The shelters allotted two 2-h blocks per day. In our initial protocol, we planned on using group counseling to supplement individual counseling to increase social support among participants and increase retention. However, we found that homeless individuals in these particular studies were not interested in meeting in groups. In addition, scheduling groups around the transient individuals in this population made finding appropriate times for all the participants in the group difficult. In addition, it was sometimes challenging finding a private space at the shelters to conduct counseling; as a result, participants had to be rescheduled within their specific time window.

Designing a feasible and practical intervention to accommodate missed visits and maintain a flexible schedule is key to maximizing adherence to the intervention protocol. It is important to keep in mind that delaying treatment initiation may contribute to attrition. For example, retention increased when NRT was administered at the baseline visit rather than waiting to the 1-week visit.

### Retention

In PTQI and PTQII we collected multiple contact sources from the participants, this allowed the team to reach those with government-issued phones, no phone number or contact information; or extremely transient individuals. Community mobilizers (see below) played an important role in enhancing recruitment and minimizing attrition. In addition, the mobilizers were tasked with the responsibility of reminding participants of the location and time of the next study appointment, and giving them reminder calls. They made reminder calls to participants during the week prior to each appointment and continued to call participants until the window for completing a given appointment closed. Calls were placed from the project office and made either to each participant’s cell phone or to the shelter identified as the most recent nighttime residence in the participant’s file. In order for study staff to retain participants it was necessary to maintain an active log of phone numbers, emails, alternate addresses, and shelter contacts for each participant. This information must be retained separately from other study documents to protect privacy.

### Compensation

In the PTQI study, the participants were compensated up to US$275 over 6 months [23]. The value of the incentives given out at any particular time was capped at US$20 with the exception of months 6 and 12 which were data collection visits in which we provided US$50. Lottery-style drawings were implemented in PTQII to bolster retention and incentivize participants to return. This type of blind incentivizing rather than a fixed-ratio compensation has been shown to increase retention [40]. Participants enrolled in both studies were compensated at every intervention and data collection visit. This incentive scheme was necessary to increase enrollment, treatment delivery, and data collection. It was also important to give compensation that was tailored to this population’s competing needs. Nonmonetary incentives were also provided; these include bus passes, tote bags, phone cards, and calendars. Calendars and pens were used with the goal that they would help with keeping study appointments.

### Adverse events

In both studies, the study coordinator ensured that adverse event documentation was thoroughly reviewed by the principal investigator (a practicing board-certified family physician). In addition, participants were given the study office phone number (24-h coverage) to contact study staff and/or the investigators to report adverse events. The study followed the NIH guidelines for reporting adverse events to the Institutional Review Board. Any problems needing medical attention were referred to the licensed provider and clinics, which are federally qualified community health centers that provide medical care and social services for homeless persons. The study was discontinued if participants became pregnant or developed a contraindication to continuing in the study. Documentation of adverse events is especially important in this population due to the high prevalence of medical and psychiatric health problems. In order to accurately assess adverse events, participants were asked questions to elicit responses concerning recent hospital visits and any other pertinent events or information at every visit.

#### Data collection and management

In PTQI and PTQII, screening for alcohol intoxication was conducted before each appointment using with Alco-Sensor III breathalyzers (Intoximeters, Inc., St. Louis, MO, USA) testing [41]. Any participant who showed a blood alcohol level (BAL) reading of 0.08 or above was unable to participate in activities for that visit. Some other studies in the homeless have used a cutoff mark of a BAL of 0.05 [42]. A criterion for proceeding or rescheduling data collection visits due to intoxication or lack of sleep was required. Study staff used the Short Blessed Test [30] to evaluate cognitive impairment prior to data collection at all visits. Breathalyzer screens were conducted before each appointment. Timing of data collection can have an important impact on participant recruitment and retention. For example, baseline data collection can occur at the time of enrollment (which improves follow-up after enrollment) or some days later (which may compromise retention). Biospecimen collection protocols should be as simple as possible and can be a sensitive requirement due to concern about using biospecimens for drug monitoring.

##### Opioids and methadone use

By design, participants in the PTQI and PTQII studies were tobacco and alcohol users. In addition, those with current drug dependence were allowed in the study with the exception of dependence on prescription drugs (e.g., opioids) due to the complexities of managing and monitoring their treatments with providers. Research staff were trained to assess alcohol and drug-related adverse events and consult with study investigators for further evaluation and a determination regarding study continuation.

#### Staffing issues

##### Appropriate licensing of professionals

The PHQ-9 was used as the tool to screen for depression and suicidal ideation in both studies. Then the CSSR-S [31] was used if the patient endorsed suicidal ideation. The CSSR-S is a six-question instrument for evaluating the severity of suicidal ideation, intent, and plan that can be used by unlicensed research staff who have been through the appropriate training [31]. Staff then documented the answers and facilitated the appropriate follow-up and referral to a mental health professional. Study staff were trained by the Columbia University training module on the CSSR-S. Thus, both study staff and shelter staff could accurately administer and determine suicidality with uniform information. The research staff worked in pairs so that one person could monitor an at-risk participant while another was seeking help. It is critical that research staff only administer screening tools and provide treatments that are appropriate to their licensure and scope of practice. Due to the clinical challenges in homeless populations it may be tempting, in an effort to be helpful, to expand that scope but this requires special attention to training and supervision. For example, there were many discussions about which screening tool to use to best assess suicidality in the participants and make appropriate referrals. Similar to other assessments, the screening tool used would have to be widely accepted, easy to work with, but also compatible with the level of licensure of both the study staff and shelter staff involved in the assessment of the participant.

##### Ethics and cultural-sensitivity training

Research staff in both studies received traditional research training that included protecting sensitive information regarding drug and alcohol use, adequate Health Insurance Portability and Accountability Act of 1996 (HIPAA) and Collaborative Institutional Training Initiative (CITI) research training, sexual harassment prevention training, protection of participants reporting sexual harassment and administering unbiased questionnaires [25, 26]. It was important to train staff in a variety of additional day-to-day challenges that this population frequently encounters and their relationship to professional boundaries. For example, clarifying how to discuss participant incentives, and the practice of offering study remuneration may be perceived or interpreted as coercive. Formalizing the training and practice of setting professional boundaries was also important given the unique work setting (see below). Studies designed for homeless populations must keep in mind that participants in shelters are in their home setting and staff are likely to see them on a daily basis. Furthermore, staff should be conscious of their professional roles during all interactions with participants.

##### Interactions with participants and professional boundaries

It was necessary to provide sexual harassment training to our staff in both studies. This training was administered by an independent certified group and focused on ways to respond appropriately and factors to consider gauging whether a participant or potential participant was acting inappropriately. In addition to online educational tutorials, staff were given role-play scenarios to equip staff with the wherewithal to appropriately identify forms of sexual harassment and to properly handle sensitive situations. Special reminders were given that under no circumstance should study staff engage in any form of personal relationship with a research participant. In order to ensure that staff acted professionally in all situations, training protocols covered topics such as proximity to participants, how to address participants using last names, and using appropriate facial expression and body gestures towards participants. All study staff were trained to sit across the table from the participants to establish distance and a professional demeanor. Only formal greetings and handshakes were permitted; other physical greetings (e.g., hugs) were off limits and staff were trained on how to respectfully explain the reasons for this boundary.

##### Special study personnel roles: community mobilizer

Community mobilizers in both studies were trained individuals who were formerly homeless or familiar with issues in homeless populations [23]. These mobilizers assisted with recruitment, coordination, and follow-up of the participants. They also worked to build a bridge of trust between the study staff and the participants. They translated the technicalities of the research information into an unbiased delivery that participants could more readily understand. During the follow-up phase of the study, they were responsible for reminding participants of the location and time of their next appointment, administering surveys, and handing out nicotine replacement therapy refills. In our PTQI study, lack of time and forgetting were the most commonly cited reasons for missing appointments [23]. Other reasons that participants gave for missing their appointments included miscommunications and competing needs such as lack of transportation. Respondents in PTQI reported that phone calls were the most helpful type of reminder [23]. Each mobilizer carried a log that documented their contact with participants including when and where the contact occurred and the nature of the contact. They were also given a protocol to follow and a checklist of activities that occurred during each contact.

## Discussion

This paper reports on the complexities of conducting two RCTs of smoking interventions conducted among persons who are homeless. The value and importance of developing protocols that support the inclusion of homeless populations in trials cannot be overemphasized. There are key research questions to address but the more inclusive a study is, the more planning is needed in order to support the participation. Homeless individuals constantly deal with problems that may make it particularly difficult to participate in research protocols, including the need for negotiating daily access to food, clothing, and shelter. Research protocols are often ill prepared to address the daily challenges that homeless individuals encounter which makes it difficult to execute clinical trials in this underserved community. Many clinical trials are avoided in this high-risk population due to concerns about safety, recruitment, and retention. Designing a comprehensive study protocol that addresses these concerns can increase the effectiveness of executing a controlled trial in homeless populations.

This study has some limitations. First, the two studies were conducted at a single metropolitan area in the upper Midwest of the United States, and the challenges faced may not generalize to all homeless persons. Secondly, limited data on this topic are available from other randomized clinical trials. Therefore, the research experience is descriptive and the recommendations are based on the experience of the research team at a single institution conducting research in this community over the past decade.

## Conclusion

There is a large population of homeless individuals in the United States who are not typically included in clinical trials, despite facing serious health challenges. In this paper we report on some of the common issues that investigators are likely to face in conducting controlled trials in the homeless population. Clinical trials are needed in this population to ascertain the best mechanisms to provide it with adequate health care. Most approaches to conducting clinical trial methods are not adequate to handle the unique challenges in the homeless population. The strategies implemented in the studies discussed in this paper may assist other researchers in designing robust protocols enrolling persons who are homeless.

## Abbreviations

AUDIT: Alcohol Use Disorders Identification Test
BAL: Blood alcohol level
CAB: Community Advisory Board
CBT: Cognitive behavioral therapy
EMS: Emergency medical services
HIPAA: Health Insurance Portability and Accountability Act of 1996
IntS + A: Integrated Intensive Smoking and Intensive Alcohol Counseling
IS: Intensive smoking
M.I.N.I.: Mini International Neuropsychiatric Interview
MI: Motivational interviewing
NIH: National Institute of Health
NRT: Nicotine replacement therapy
PHQ-9: Patient Health Questionnaire-9
PTQ: Power to Quit
UC: Usual care
